# Bone Marrow Oxidative Stress and Acquired Lineage-Specific Genotoxicity in Hematopoietic Stem/Progenitor Cells Exposed to 1,4-Benzoquinone †

**DOI:** 10.3390/ijerph17165865

**Published:** 2020-08-13

**Authors:** Ramya Dewi Mathialagan, Zariyantey Abd Hamid, Qing Min Ng, Nor Fadilah Rajab, Salwati Shuib, Siti Razila Binti Abdul Razak

**Affiliations:** 1 Biomedical Science Programme and Centre for Diagnostic, Therapeutic and Investigative Studies, Faculty of Health Sciences, Universiti Kebangsaan Malaysia, Kuala Lumpur 53000, Malaysia; soniamirza05@hotmail.com (R.D.M.); qingmin136@gmail.com (Q.M.N.); 2 Biomedical Science Programme and Center for Healthy Ageing & Wellness, Faculty of Health Sciences, Universiti Kebangsaan Malaysia, Kuala Lumpur 53000, Malaysia; nfadilah@ukm.edu.my; 3 Department of Pathology, Faculty of Medicine, Universiti Kebangsaan Malaysia, Jalan Yaacob Latif, Bandar Tun Razak, Cheras, Kuala Lumpur 56000, Malaysia; salwati@ppukm.ukm.edu.my; 4 Oncological and Radiological Sciences Cluster, Advanced Medical & Dental Institute, Universiti Sains Malaysia, Kepala Batas Bertam, Pulau Pinang 13200, Malaysia; sitirazila@usm.my

**Keywords:** 1,4-BQ, oxidative stress, genotoxicity, hematopoietic stem/progenitor cells, lineage-directed strategy

## Abstract

Hematopoietic stem/progenitor cells (HSPCs) are susceptible to benzene-induced genotoxicity. However, little is known about the mechanism of DNA damage response affecting lineage-committed progenitors for myeloid, erythroid, and lymphoid. Here, we investigated the genotoxicity of a benzene metabolite, 1,4-benzoquinone (1,4-BQ), in HSPCs using oxidative stress and lineage-directed approaches. Mouse bone marrow cells (BMCs) were exposed to 1,4-BQ (1.25–12 μM) for 24 h, followed by oxidative stress and genotoxicity assessments. Then, the genotoxicity of 1,4-BQ in lineage-committed progenitors was evaluated using colony forming cell assay following 7–14 days of culture. 1,4-BQ exposure causes significant decreases (*p* < 0.05) in glutathione level and superoxide dismutase activity, along with significant increases (*p* < 0.05) in levels of malondialdehyde and protein carbonyls. 1,4-BQ exposure induces DNA damage in BMCs by significantly (*p* < 0.05) increased percentages of DNA in tail at 7 and 12 μM and tail moment at 12 μM. We found crucial differences in genotoxic susceptibility based on percentages of DNA in tail between lineage-committed progenitors. Myeloid and pre-B lymphoid progenitors appeared to acquire significant DNA damage as compared with the control starting from a low concentration of 1,4-BQ exposure (2.5 µM). In contrast, the erythroid progenitor showed significant damage as compared with the control starting at 5 µM 1,4-BQ. Meanwhile, a significant (*p* < 0.05) increase in tail moment was only notable at 7 µM and 12 µM 1,4-BQ exposure for all progenitors. Benzene could mediate hematological disorders by promoting bone marrow oxidative stress and lineage-specific genotoxicity targeting HSPCs.

## 1. Introduction

Benzene is a known hematotoxic and genotoxic agent. Human exposure to benzene can alter the homeostasis of hematopoiesis, leading to various hematological disorders and malignancies including aplastic anemia, myelodysplasia syndrome, and acute myeloid leukemia [1]. Previous studies reported that benzene exposure was able to induce genotoxicity in hematopoietic stem cells (HSCs) via chromosome aberration [1,2,3,4], DNA damage [5], and epigenetic alterations [6,7]. Despite the reported findings, the precise mechanisms underlying benzene-mediated hematological disorders and malignancies targeting HSCs niche remain elusive.

On the basis of previous reports, benzene acts via multiple modes of actions in targeting HSCs niche, causing impaired hematopoiesis. Among the reported modes of actions are induction of chromosomal aberration (CA), genomic instability, oxidative stress, DNA damage, apoptosis, error-prone DNA repair, and epigenetic impairment [8]. Benzene metabolism plays an important role in mediating hematological disorders and malignancies [9]. Benzene undergoes metabolism in the liver to produce benzene oxide via oxidation by cytochrome P4502E1. Benzene oxide is then further oxidized into reactive and toxic metabolites such as phenol, hydroquinone (HQ), catechol, and benzoquinones [10]. In the bone marrow, HQ will be further oxidized by myeloperoxidase (MPO) to produce 1,4-benzoquinone (1,4-BQ), which is known as a highly genotoxic metabolite [11].

Benzoquinones can cause oxidative DNA damage, lipid peroxidation, and strand breaks in the DNA of bone marrow cells, implicating the role of reactive oxygen species (ROS) and covalent binding in benzene-induced toxicity. Formation of DNA double strand breaks (DSBs) by ROS and other mechanisms can lead to increased mitotic recombination, chromosomal translocations, and aneuploidy [3,12,13]. Such genetic consequences may result in protooncogene activation, tumor suppressor gene inactivation, gene fusions, and other deleterious changes in stem cells that can ultimately result in leukemic responses [8].

Previous epidemiology studies have reported that exposure to benzene metabolite 1,4-BQ causes DNA damage in mice [14]. Furthermore, 1,4-BQ exposed-CD34^+^ cells demonstrated a higher percentage of micronuclei as compared with the control group [15]. In addition, benzene has been reported to impose genotoxic effects and promote greater DNA damage in workers with occupational exposure, as compared with the control [16]. Despite reports concerning genotoxic effects of benzene targeting bone marrow compartments, these reports mainly focus on the effect of benzene exposure on single subpopulation of cells in bone marrow and hematopoietic system. Among the most common conventional population of cells being investigated were blood lymphocytes, CD 34 ^+^, and Sca-1^+^ cells, which do not represent the comprehensive subpopulation of lineage-specific cells that are crucial for establishment and homeostasis of HSCs niche.

HSCs niche in bone marrow is a complex microenvironment comprised of HSCs subpopulation and lineage-committed hematopoietic progenitors that are vital for the maintenance of haematopoiesis [17,18]. HSCs and hematopoietic progenitors are precursor cells that are capable to self-renew and differentiate to form a particular type of new blood cell under the regulation of intrinsic factors (e.g., signaling pathway or transcription factors) and extrinsic factors (cytokines or growth factors) [19]. Therefore, balanced regulation of self-renewal and differentiation activities in HSCs and progenitor cells is important as any perturbation to these activities can lead to haematopoiesis impairment and subsequent cellular transformation and malignancies [20].

Previously, our group has demonstrated a lineage-dependent response as emerging evidence linking hematopoietic stem/progenitor cells (HSPCs) of different lineages with benzene toxicity [21,22]. These studies demonstrated the role of lineage-specificity in governing differential cytotoxicity effects of benzene in targeting HSC niche. In bone marrow, 1,4-benzoquinone (1,4-BQ) has been reported as the most reactive benzene metabolite to cause bone marrow toxicity and hematotoxicity via oxidative stress and genotoxicity mechanisms [23]. Because the genotoxic agents can induce genomic abnormalities, such alterations may result in dysregulated or dysfunctional mRNA and proteins, leading to various cellular abnormalities such as alterations in cell cycle as well as malignant transformation. At present, the best and most efficient approach to monitor genotoxicity effects of benzene targeting subpopulation of hematopoietic progenitor of various lineages is not yet clear and requires further translational investigation, particularly concerning relevant in vitro models. Therefore, a novel strategy for genotoxicity monitoring will be essential.

Thus, in this study, we focused on the toxicity of 1,4-BQ to uncover the potential mechanism underlying benzene-induced bone marrow oxidative stress and subsequent genotoxicity effect targeting hematopoietic stem/progenitor cells (HSPCs) via DNA damage study of specific hematopoietic lineages-committed progenitors. The use of a toxicogenomic approach combined with the lineage-directed strategy is vital in order to understand the mechanism of benzene-induced hematological disorders and malignancies through oxidative stress and genotoxicity targeting HSCs niche. Meanwhile, the use of colony forming cells (CFCs) assay could offer a biological platform to support the specific detection of genomic changes in parental hematopoietic progenitor cell, which is vital to provide complementary information that may contribute to later risk associated with benzene-induce hematotoxicity and leukemogenesis targeting HPSCs of different lineages.

## 2. Materials and Method

### 2.1. Experimental Design

**Figure 1:**
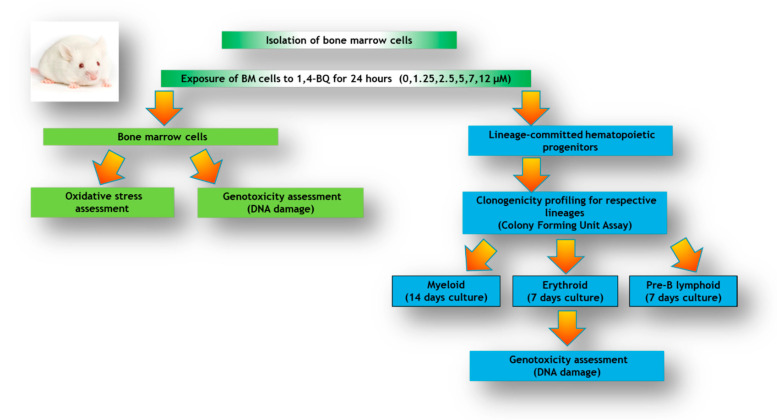
Research design illustrating the experimental workflow involving analysis of bone marrow (BM) cells and lineage-committed hematopoietic progenitors comprised of myeloid, erythroid, and pre-B lymphoid treated with 1,4-BQ for 24 h.

In this study, we investigated the oxidative stress profiles on bone marrow followed by DNA damage response of bone marrow cells and subsequent genotoxic consequences in hematopoietic progenitors comprised of myeloid, erythroid, and pre-B lymphoid lineages. The research design for the current study is shown in Figure 1.

### 2.2. Isolation of Mouse Bone Marrow Cells and Cell Culture Condition

The procedures that involve the use of mice (*Mus musculus musculus*) were approved by the Animal Ethics Committee of the Universiti Kebangsaan Malaysia (UKMAEC, Kuala Lumpur, Malaysia) with the approval number FSK/2016/ZARIYANTEY/23-NOV/814-NOV-2016-JULY-2019-AR-CAT2. Male imprinting control region-strained (ICR) mice were purchased from the Laboratory Animal Resource Unit, Faculty of Medicine, Universiti Kebangsaan Malaysia, Kuala Lumpur, Malaysia. The ICR mice were used in the current study to maintain a similar phenotype of laboratory animal being used for isolation of bone marrow cells and HSPCs as reported in previous studies by our team [21,22]. Bone marrow cells (BMCs) were obtained through femur and tibia flushing from 10-week-old adult male mice [14,24]. Collected BMCs were filtered through a 40 μM nylon mesh (BD Biosciences, San Jose, CA, USA) and suspended in complete cell culture media consisting of DMEM (Dulbecco’s Modified Eagle Medium) supplemented with 10% FBS, 1% penicillin/streptomycin, 100 ng/mL stem cells factor (SCF), 10 ng/mL interleukin-6 (IL-6), and 5 ng/mL interleukin-3 (IL-3) [21,25,26]. Cells were then maintained overnight in a humidified incubator at 37 °C and 5% CO_2_.

### 2.3. 1,4-BQ Exposure

Stock solution of 50 mM 1,4-BQ was freshly prepared in a phosphate buffer solution. Mouse BMCs (1 × 10^6^ cells/mL) were exposed to 0, 1.25, 2.5, 5, 7, and 12 μM of 1,4-BQ for 24 h at 37 °C and 5% CO_2_ [21]. The dose selection was based on the MTT (3-(4,5-Dimethylthiazol-2-yl)-2,5-diphenyltetrazolium bromidefor) assay in determination of 1,4-BQ cytotoxicity performed previously, in which the inhibition concentration of 10% cell viability (IC_10_), inhibition concentration of 25% cell viability (IC_25_), and inhibition concentration of 50% cell viability (IC_50_) were 5, 7, and 12 μM, respectively. Doses below 5µM were used as 1,4-BQ exposure at non-cytotoxic concentrations (1.25 and 2.5 μM), as described in previous study [22].

### 2.4. Determination of Bone Marrow Oxidative Stress Profiles

Bone marrow oxidative stress profiles were studied using panels of antioxidants, namely glutathione (GSH) and superoxide dismutase (SOD), along with markers for oxidative damage comprising malondialdehyde (MDA) and protein carbonyls (PCs). Briefly, cell lysate was prepared from the cell suspension after 24 h of 1,4-BQ exposure and protein levels were determined with Bradford assay prior to antioxidant analysis. Quantification of GSH involves oxidation of GSH by the sulfhydryl reagent 5,5′-dithio-bis (2-nitrobenzoic acid) (DTNB) to form the yellow derivative 5′-thio-2-nitrobenzoic acid (TNB) that was measurable at 415 nm [27]. GSH levels were expressed as mmol/mg. As for determination of SOD activity, the activity was assayed based on the inhibition of nitroblue-tetrazolium (NBT) reduction [28]. The reduction of NBT by superoxide radicals to blue coloured formazan was read at 560 nm and expressed as U/min/mg. Then, the level of MDA was assessed following the reaction of one molecule of MDA with two molecules of thiobarbituric acid (TBA) to form a chromophore via extreme boiling (DNPH) in which the reaction protein was precipitated and excessive DNPH was removed at 100 °C, followed by measurement at 532 nm [29]. MDA level was expressed as nmol/g. Determination of PC level is done based on the reaction between protein oxidation and 2, 4-dinitrofenalhidrazin [30]. The protein pellet was dissolved in urea solution at 37 °C and the supernatant was measured at 360 nm wavelength in spectrophotometric assay. PC level was expressed as nmol/g.

### 2.5. Colony Forming Cells Assay

The colony forming cells (CFCs) assay was performed following the manufacturer’s protocol (Stem Cells Technologies). Three types of specialized methylcellulose culture media enriched with specialized combinations of growth factors were used to support the growth of respective lineage-committed hematopoietic progenitors comprising erythroid, myeloid, and pre-B lymphoid lineages. Erythroid progenitors comprised of colony-forming unit-erythroid (CFU-E) and mature burst-forming unit-erythroid (BFU-E) were enriched following 7 days of culture using MethoCult media #03334 supplemented with recombinant human (rh) insulin, human transferrin (iron-saturated), and rh erythropoietin (EPO) specific for detection of CFU-E. Meanwhile, for myeloid progenitors comprised of colony-forming units for granulocyte macrophage (CFU-GM), granulocyte (CFU-G), and macrophage (CFU-M) were enriched using MethoCult media #03534 containing recombinant mouse (rm) stem cell factor (SCF), rm interleukin 3 (IL-3), rh interleukin 6 (IL-6), rh insulin, and human transferrin (iron-saturated) after 14 days of culture. Pre-B lymphoid progenitors comprised of colony-forming unit-pre-B (CFU-pre-B) were enriched following 7 days of culture using MethoCult #03630 supplemented with rh interleukin 7 (IL-7). In brief, after 24 h of treatment, the harvested BMCs were centrifuged at 2500 rpm for 7 min and supernatants were removed. The cells were resuspended in fresh media, and cell count was performed. A total of 1 × 10^5^, 2 × 10^4^, and 5 × 10^4^ cells were added into 1 mL of specialized methylcellulose media for culture of erythroid, myeloid, and pre-B lymphoid progenitors, respectively. Cultures were incubated at 37 °C with 5% CO_2_ for respective days prior to colony analysis for progenitor assessment. The colonies for respective progenitor were observed under an inverted microscope and harvested for downstream DNA damage analysis using alkaline comet assay.

### 2.6. Evaluation of DNA Damage by Alkaline Comet Assay

In this study, we investigated the DNA damage response of bone marrow cells following single exposure to 1,4-BQ and subsequent genotoxic consequences in hematopoietic progenitors through which progenitors for myeloid, erythroid, and pre-B lymphoid lineages were assessed for acquired DNA damage. DNA damage was determined using alkaline comet assay following protocol as described [31]. Briefly, after 24 h of 1,4-BQ exposure, bone marrow cells were harvested for the following analysis. One is intended for immediate comet assay and the other for CFC assay to grow lineage-committed hematopoietic progenitors, and sampling was carried out immediately after incubation period for further comet assay study.

Prior to comet assay, sample preparation was accomplished using the following procedure. The harvested cells were washed twice in Ca^2+^- and Mg^2+^-free PBS by centrifugation at 2500 rpm for 5 min at 4 °C. Then, cells were mixed with 0.6% low melting point agarose (LMA) and spread on a frosted microscopic slide pre-coated with 0.6% normal agarose. Slides were then left cooled on ice for 10–15 min to solidify the agarose gel. Next, cells were lysed by immersing the slides into a coplin jar filled with lysis buffer (2.5 M NaCl, 100 mM Na_2_-EDTA (Ethylenediaminetetraacetic acid), 10 mM Tris, and 1% Triton X-100) overnight at 4 °C. Then, slides were placed in electrophoresis buffer for 20 min to break the formation of double strands in DNA. The slides were then electrophoresed at 3000 mA, 25 V for 20 min and rinsed with neutralization buffer three times. Next, slides were stained with ethidium bromide (10 µg/mL) in protection from light and were observed under the fluorescence microscope using 590 nm excitation filter. The percentage of DNA in tail and tail moment of 100 cells per slide was calculated using the CometScore^TM^ software (https://www.bioz.com/search/comet%20score%20software).

### 2.7. Statistical Analysis

Each experiment was conducted in triplicate, and the data are presented as the means ± standard error of mean (SEM). Statistical analysis was conducted using GraphPad Prism 7 and the level of significance used for all statistical tests was *p* < 0.05. An analysis of variance (ANOVA) test was performed to compare the parameters in each treatment group and in the control group. Dunnett’s *t*-test (assuming equal variance) was used to identify treatment groups that differed from the control group.

## 3. Results

### 3.1. Effect of 1,4-BQ Exposure on Bone Marrow Oxidative Stress Profiles

**Figure 2:**
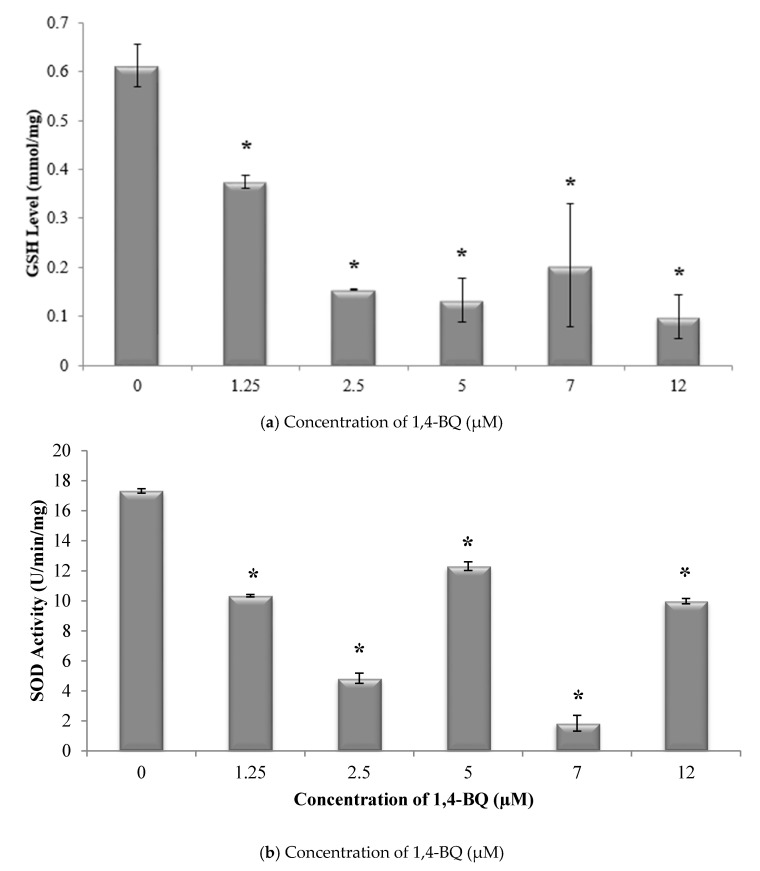
Effect of 1,4-benzoquinone (1,4-BQ) exposure on (**a**) glutathione (GSH) levels and (**b**) superoxide dismutase (SOD) activity in mouse bone marrow cells after 24 h treatment with 1,4-BQ. Data are presented as the mean ± S.E.M. (*n* = 3). * *p* < 0.05 compared with the control group.

**Figure 3:**
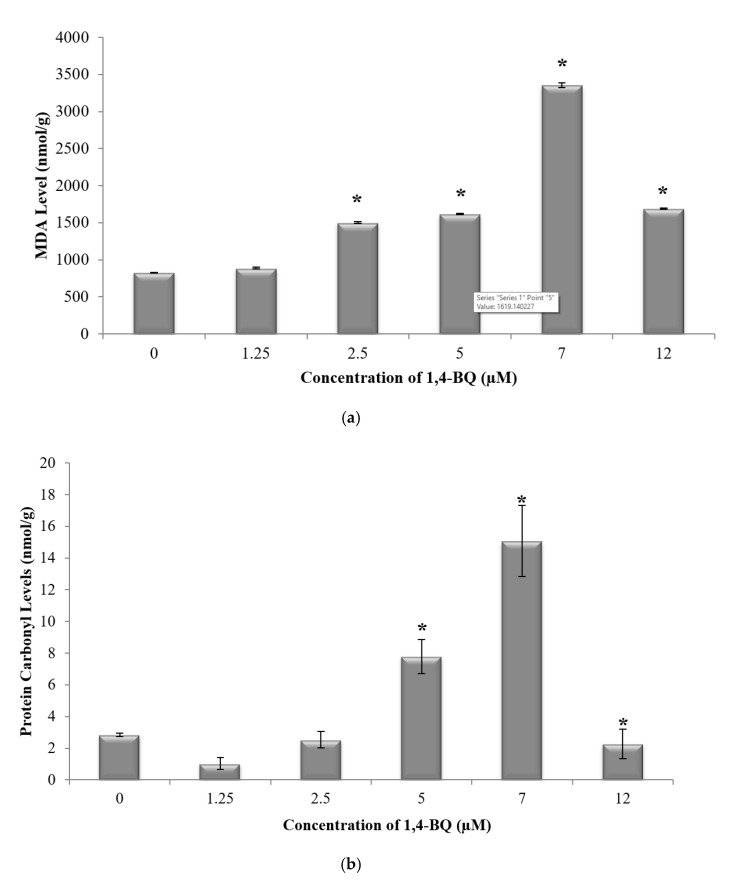
Effect of 1,4-BQ exposure on (**a**) malonaldehyde (MDA) and (**b**) protein carbonyl levels in mouse bone marrow cells after 24 h treatment with 1,4-BQ. Data are presented as the mean ± S.E.M. (*n* = 3). * *p* < 0.05 compared with the control group.

Glutathione (GSH) and superoxide dismutase (SOD) are the most important antioxidative enzymes. GSH is a tripeptide that contains L-cysteine, L-glutamic acid, and glycine, which prevents cell damage caused by reactive oxygen species such as free radicals and peroxides [27]. Meanwhile, SOD catalyzes the dismutation of the superoxide anion into hydrogen peroxide and molecular oxygen. Both are essential for evaluating the redox and detoxification status of the cells and tissues against oxidative and free radicals mediated cell injury [28]. Lipid peroxidation has been established as a major mechanism of cellular injury in many biological systems of plant and animal origin. Lipid peroxides are themselves unstable, and further react to form malonaldehyde (MDA) [29]. The MDA result of lipid peroxidation has become one of the most widely reported analytes for the purpose of estimating oxidative stress effects on lipids. In addition, oxidative stress has been shown to increase protein oxidation. Protein carbonyl content is widely used as both a marker for oxidative stress and a measure of oxidative damage. It is a widely used method for the determination of protein carbonylation utilizing the reaction of carbonyl groups with 2,4-dinitrophenylhydrazine (DNPH) to form protein-bound 2,4-dinitrophenylhydrazones [30]. Thus, these markers were assessed to determine the effect of 1,4-BQ exposure on the oxidative stress status of exposed bone marrow cells. A significant reduction (*p* < 0.05) in GSH level (Figure 2a) and SOD activity (Figure 2b) was noted following exposure to 1,4-BQ at all tested concentrations as compared with the untreated control. Meanwhile, a significant increase (*p* < 0.05) in malonaldehyde level, the metabolic product of lipid peroxidation, was noted following exposure to 1,4-BQ at 2.5 μM, 5 μM, 7 μM, and 12 μM as compared with the untreated control (Figure 3a). However, a significant increase (*p* < 0.05) in protein carbonyl level was only notable at 5 μM and 7 μM of 1,4-BQ as compared with the untreated control group (Figure 3b).

### 3.2. DNA Damage Profile of 1,4-BQ-Exposed Mouse Bone Marrow Cells

**Figure 4:**
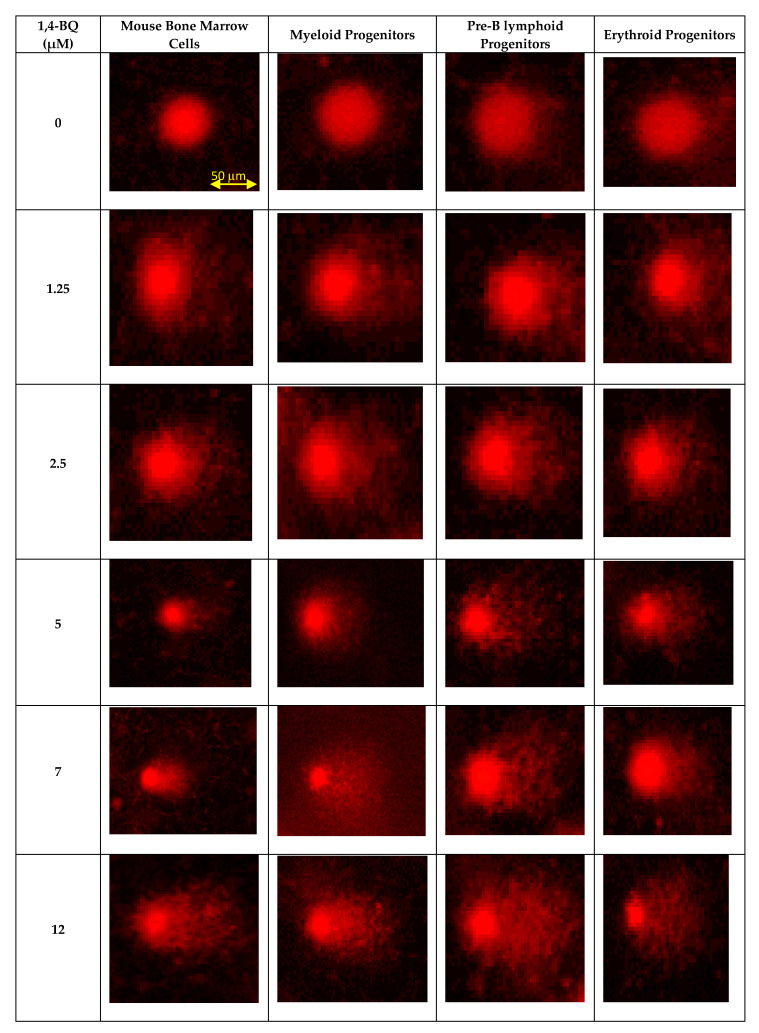
Photomicrographs of comet assay indicate status of DNA damage following 1,4-BQ exposure at various concentrations in bone marrow cells and hematopoietic progenitors for myeloid, pre-B lymphoid, and erythroid lineages. All the photographs are in the scale of 50 µm.

**Figure 5:**
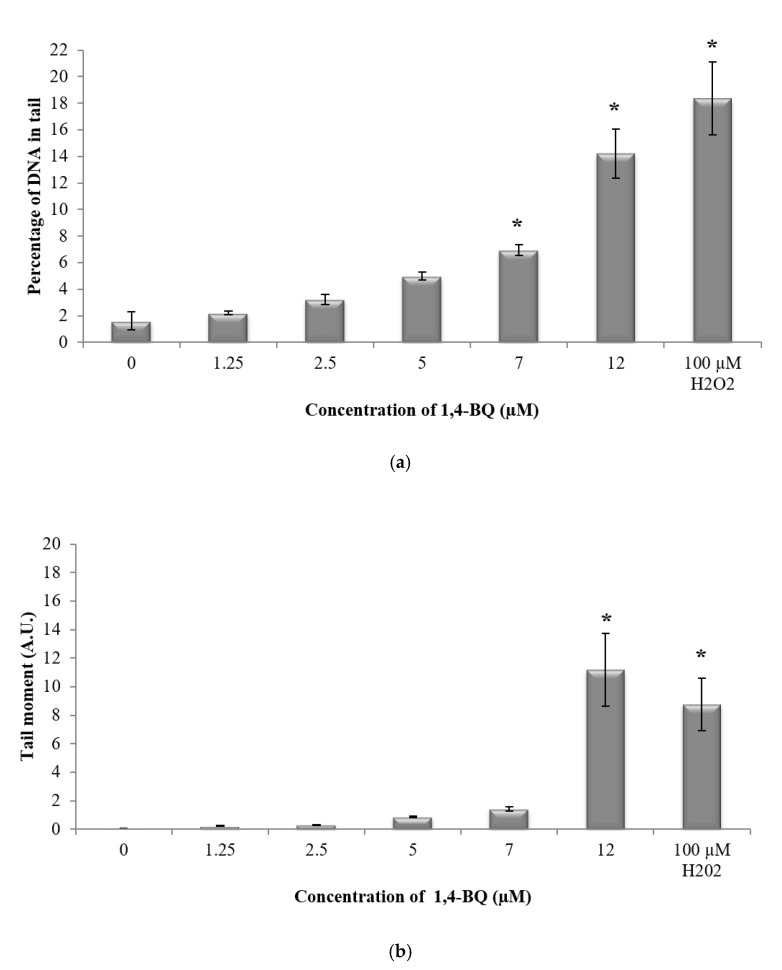
Effect of 1,4-BQ exposure on DNA damage profiles of exposed mouse bone marrow cells after 24 h exposure indicated by the (**a**) percentage of DNA in tail and (**b**) tail moment. Data are presented as the mean ± S.E.M. (*n* = 3). * *p* < 0.05 compared with the untreated control group.

In this study, alkaline comet assay was carried out to measure DNA strand breaks to evaluate the genotoxic effect of 1,4-BQ at the individual cell level [31]. The DNA damage profile of 1,4-BQ-exposed mouse BMCs was evaluated immediately after 24 h exposure at various concentrations of 1,4-BQ (1.25, 2.5, 5, 7, and 12 μM). Hydrogen peroxide with the concentration of 100 mM was used in this study as a positive control. Exposure to 1,4-BQ caused DNA damage in mouse bone marrow cells in a dose-dependent manner. Figure 4 shows the representative microscopic appearances of comet assay showing the DNA distribution in bone marrow cells. A significant (*p* < 0.05) increase in the percentage of DNA in tail was observed at 1,4-BQ exposure at 7 μM (6.93%) as compared with the untreated control group (1.62%) (Figure 5a). Similarly, tail moment of bone marrow cells was observed to increase significantly at 1,4-BQ concentration of 12 μM (11.17 A. U) as compared with the control group (0.04 A. U) (Figure 5b).

### 3.3. DNA Damage Profiles of 1,4-BQ-Exposed Lineage-Committed Hematopoietic Progenitors

**Figure 6:**
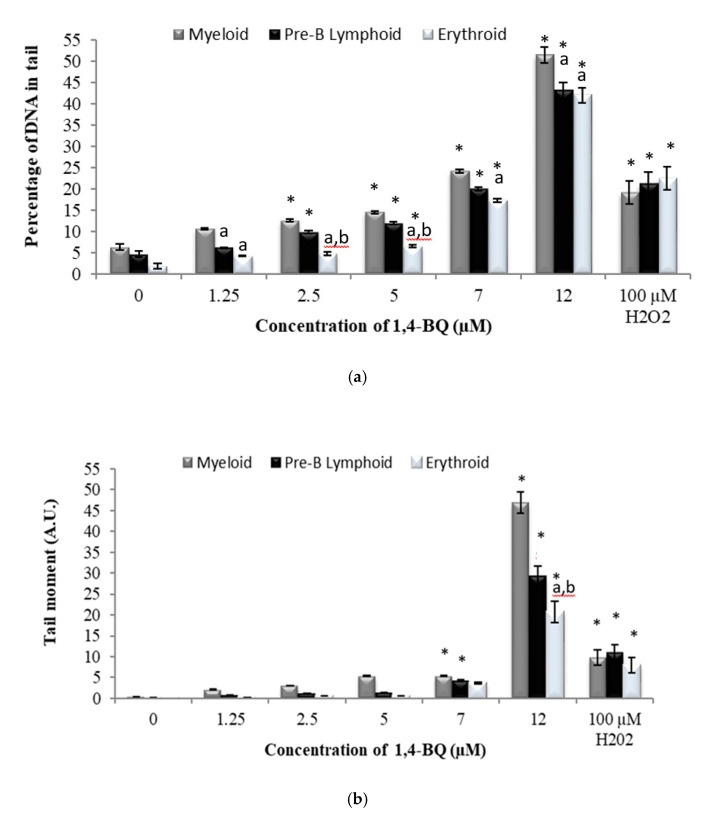
Effect of 1,4-BQ exposure for 24 h on the DNA damage profiles of lineage-committed hematopoietic progenitor cells indicated by (**a**) percentage of DNA in tail and (**b**) tail moment, which were analysed after 14 days of colony forming cells culture for myeloid, and 7 days for pre-B lymphoid and erythroid progenitors. Data are presented as the mean ± S.E.M. (*n* = 3) and * represents significance compared with the untreated control group within the same progenitors (*p* < 0.05), a represents significance compared with myeloid progenitors (*p* < 0.05), and b represents significance compared with pre-B lymphoid progenitors (*p* < 0.05).

The DNA damage profile for lineage-committed progenitors was evaluated following colony harvest from CFC assay on day 7 for pre-B lymphoid and erythroid progenitors and on day 14 for myeloid progenitor. CFC assay is the most widely used in vitro assay in the study of HSPCs. The assay allows measurement of the proliferation and differentiation ability of an individual hematopoietic progenitor to form colonies in a semi-solid media in response to cytokine stimulation. Thus, the assay is used in this study as a platform to determine the genotoxic effect of 1,4-BQ exposure targeting HSPCs of respective erythroid, myeloid, and pre-B lymphoid lineages. Hydrogen peroxide with the concentration of 100 mM was used in this study as a positive control. Exposure to 1,4-BQ induced DNA damage in lineage-committed hematopoietic progenitors (myeloid, pre-B lymphoid, and erythroid) via concentration and lineage-dependent manners. A significant increase in percentage of DNA in tail (*p* < 0.05) in myeloid progenitor cells was observed upon 1,4-BQ exposure at 2.5 µM, 5 µM, 7 µM, and 12 µM (Figure 6a) as compared with the untreated control group. However, a significant increase in tail moment (*p* < 0.05) was only notable at 7 µM and 12 µM 1,4-BQ exposure as compared with the untreated control group for myeloid progenitor cells (Figure 6b). In addition, a significant increase in percentage of DNA in tails (*p* < 0.05) was observed at 2.5 µM, 5 µM, 7 µM, and 12 µM upon 1,4-BQ exposure in pre-B lymphoid progenitor cells (Figure 6a). Meanwhile, a significant (*p* < 0.05) increase in tail moment was observed only at 7 µM and 12 µM as compared with the untreated control group for lymphoid progenitor cells (Figure 6b). In addition to erythroid progenitor cells, a significant increase in percentage of DNA in tail (*p* < 0.05) was observed only at 5 µM, 7 µM, and 12 µM 1,4-BQ exposure (Figure 6a). Meanwhile, a significant (*p* < 0.05) increase in tail moment was observed only at 7 µM and 12 µM as compared with the untreated control group for erythroid progenitor cells (Figure 6b). Meanwhile, comparison of DNA damage in between progenitors (Figure 6) shows that myeloid progenitor possessed higher percentage of DNA damage involving both the percentage of DNA in tail and tail moment as compared with pre-B lymphoid and erythroid progenitors. Figure 4 shows the representative microscopic appearances of comet assay showing DNA distribution in myeloid, pre-B lymphoid, and erythroid progenitors.

## 4. Discussion

In the present study, exposure to 1,4-BQ caused oxidative stress in mouse bone marrow cells at all tested concentrations, including those at non-cytotoxic concentrations (1.25 μM and 2.5 μM). This finding could further justify the ability of 1,4-BQ to induce bone marrow injury even at non-cytotoxic concentrations. It was notable that MDA levels increased upon 1,4-BQ exposure in mouse bone marrow cells, indicating the ability of 1,4-BQ to cause oxidative lesions via lipid peroxidation. This finding is in line with previous studies reporting that benzene exposure induced oxidative stress by generating increased reactive oxygen species (ROS) [32,33,34,35].

In addition to lipids, free radicals can also react with proteins with protein carbonyl (PC) production, which are among the established biomarkers to indicate protein oxidation [36,37]. In this study, we detected an increase in PC levels in 1,4-BQ treated groups as compared with the untreated control group. Redox cycling conversion of hydroquinone and benzoquinone emits reactive oxygen species and hydroxide ions that are able to break the peptide chain, alter the protein electrical charge, cross-link proteins and oxidize specific amino acids, all of which can cause oxidative DNA damage in hematopoietic stem cells [8]. Thus, this finding shows that benzene metabolite can cause oxidative DNA damage via the ROS-induced oxidative stress pathway involving lipid peroxidation and protein oxidation. In addition, our study noted that lipid peroxidation was significantly induced starting at a lower concentration (5 µM) of 1,4-BQ exposure as compared with protein carbonyl, which was significantly elevated only at 7 µM of 1,4-BQ exposure. The notable finding may indicate that bone marrow cells are more susceptible to cellular membrane damage acquired by lipid peroxidation than the cellular damage of proteins via protein oxidation.

Meanwhile, oxidative damage products (MDA and PC) in mouse bone marrow cells exhibited a tendency to decrease at 12 μM as compared with 7 μM. This is believed to be because of greater cytotoxicity and apoptosis in mouse bone marrow cells that occur following exposure to 1,4-BQ at 12 μM, as 1,4-BQ is very reactive at higher concentrations, leading to cell membrane damage and apoptosis [32,38]. Hence, 1,4-BQ caused greater cell death than oxidative damage at a higher concentration of 12 μM. These findings are in agreement with the previous study of [39], who reported that micronuclei frequency in human lymphocytes was not significantly increased at higher concentrations of 1,4-BQ exposure. Therefore, the extracellular exposure of a high concentration of 1,4-BQ caused cytotoxicity with reduced oxidative lesions as most cells undergo cell death modulated by the apoptotic pathway.

Physiologically, oxidative stress is a result of increased ROS and/or reactive nitrogen species (RNS) and impaired activities of antioxidant defence enzymes [35,36]. There is growing evidence that benzene metabolism involves redox cycling producing reactive oxygen species (ROS), leading to reduced gluthathione (GSH) and causing further oxidative stress in bone marrow cells [37,38,39]. This may be owing to the nature of bone marrow cells, which are highly expressed with enzyme myeloperoxidase (MPO), whereby the MPO has been reported to play a significant role in benzene-induced oxidative stress via its ability to oxidize HQ to highly reactive BQ, leading to the production of superoxide and other radicals in the bone marrow compartment [23]. In the present study, we observed reduced superoxide dismutase (SOD) activity and GSH level upon 1,4-BQ exposure. This finding further illustrates the ability of benzene metabolite 1,4-BQ to suppress the antioxidant enzymes by generating increased ROS, resulting in oxidative stress.

Benzene-induced cellular oxidative stress may damage DNA by causing strand breaks [40,41,42,43]. Our study has identified that 1,4-BQ induced DNA damage in a concentration-dependent manner for both bone marrow and HSPCs groups. Interestingly, we found crucial differences in genotoxic susceptibility among the hematopoietic progenitors and bone marrow cells in terms of the percentage of DNA in tail following exposure to 1,4-BQ. In this study, myeloid and pre-B lymphoid progenitors exhibited greater susceptibility to DNA damage as a significantly higher percentage of DNA in tail was noted starting at a lower concentration of 1,4-BQ exposure (2.5 µM) in comparison with erythroid progenitor, which exhibited significant increment starting at 5 µM of 1,4-BQ exposure. In contrast, a significantly higher percentage of DNA in tail was only evidenced in bone marrow cells following exposure to 1,4-BQ at 7 µM and 12 µM. This finding justify the importance of lineage study as it could uncover cell-type-specific effects. Meanwhile, overall comparison between respective hematopoietic progenitors indicates that myeloid progenitor showed a higher percentage of DNA in tail and tail moment than pre-B lymphoid and erythroid progenitors upon 1,4-BQ exposure.

As notable in the present study, 1,4-BQ induced DNA damage in hematopoietic progenitors via lineage-dependent. The differential genotoxic susceptibility exhibited by different lineages could be owing to the differential activity of bioactivation enzyme, namely myeloperoxidase (MPO) and detoxication enzyme, namely NAD(P)H:quinone oxidoreductase 1 (NQO1), which are acquired by these progenitors during exposure to benzene [11]. Thus, it is crucial to investigate the lineage responses in the benzene toxicity study, as this enables the detection of cell-specific response within population of hematopoietic stem cells and progenitors that are vital in maintaining haematopoiesis. In the bone marrow, HQ and catechol are converted by MPO to 1,4-benzoquinone, contributing to benzene-induced hematotoxicity. In addition to that, MPO expression was detected at a higher level in CD34^+^CD33^+^ cells, which corresponds to myeloid progenitor cells, compared with CD34^+^CD33^+^ low cells, which are more primitive blood stem cells [1,15]. Nevertheless, 1,4-BQ can be detoxified to HQ using the enzyme NQO1 [11]. Thus, the metabolic balance of these enzymes within different populations of hematopoietic cells may contribute to benzene-induced cell-specific toxicity, which deserves further investigation.

## 5. Conclusions

In conclusion, 1,4-BQ exposure increased oxidative stress in bone marrow cells via reduction of antioxidant capacity (GSH and SOD) and promotion of greater oxidative damage (MDA and protein carbonyl). Excessive oxidative stress and oxidative damage can induce genomic instability, leading to abnormal outcomes such as alterations in cell cycle and malignant transformation, including leukemogenesis. We found crucial differences in genotoxic susceptibility among the hematopoietic progenitors and bone marrow cells following exposure to 1,4-BQ. In addition, it is noteworthy that DNA damage profiles via lineage-directed study uncovers lineage-dependent genotoxicity in lineage-committed hematopoietic progenitors following exposure to 1,4-BQ. This finding could be owing to a differential adaptive response or survival mechanism acquired by these cells, which deserve further investigation. Furthermore, further studies on the status of apoptosis, oxidative damage, and status of DNA damage repair in respective lineage-committed hematopoietic progenitor cells is crucial to further understand the prolonged genotoxicity outcome affecting these progenitors in response to 1,4-BQ exposure. Overall, our findings provide useful information for biological interpretation concerning lineage-specific response and can be considered as a support tool to understand the mechanism of benzene-induced haematological disorders targeting the niche of hematopoietic stem cells and progenitors.
